# RISK OF HEART FAILURE AFTER CATHETER ABLATION VS. MEDICAL THERAPY FOR ATRIAL FIBRILLATION IN OLDER ADULTS

**DOI:** 10.1093/geroni/igac059.3044

**Published:** 2022-12-20

**Authors:** Ricardo Criado Carrero, Andrew Crawford, Ali Vaeli Zadeh, Alan Wong, Alexander Fleischhacker, Elias Collado, Joshua Larned

**Affiliations:** University of Miami/Holy Cross Hospital, Fort Lauderdale, Florida, United States; Holy Cross Health-University of Miami, Fort Lauderdale, Florida, United States; Holy Cross Health-Jim Moran Cardiovascular Research Institute, Fort Lauderdale, Florida, United States; Holy Cross Health-University of Miami, Fort Lauderdale, Florida, United States; University of Miami/Holy Cross Hospital, Fort Lauderdale, Florida, United States; Holy Cross Health-Jim Moran Cardiovascular Research Institute, Fort Lauderdale, Florida, United States; Holy Cross Health- University of Miami, Miami, Florida, United States

## Abstract

**Background:**

Age increases the risk of atrial fibrillation (AF). Catheter ablation (CA) proved being safer and superior to anti-arrhythmic drugs (AADs) in maintaining sinus rhythm and preventing readmissions. However, data on the risk of developing heart failure (HF) following each treatment in older adults is limited.

**Methods:**

A retrospective study was conducted using the PearlDiver database (PearlDiver Technologies, Fort Wayne, IN). Using ICD 9 and 10 codes, a cohort of patients without a history of HF, aged 65 to 75, with an Elixhauser Comorbidity Index (ECI) of >4 and new-onset AF (index event) was identified. The cohort was then divided into two groups based on treatment received following the index event and matched considering age, gender, ECI, and other cardiovascular diseases. Records from both groups were reviewed for the first episode of HF over 12 months following the initiation of treatment. Pearson’s chi-squared test was used to compare groups. The strength of association was reported using Risk Ratios (RR). A p-value < 0.05 was deemed significant.

**Results:**

6,446 were included in each group. The mean age, gender and ECI were indifferent. 958 (15%) of patients treated with AADs and 987 (15.3%) of patients treated with CA had a first episode of HF over a year after the treatment which was not significantly different between groups (RR=1.03, CI95% = 0.95- 1.11, p=0.49).

**Conclusion:**

The modality of treatment after the first episode of AF in older population doesn’t significantly affect the risk of HF over the first year.

